# Proposed Radiographic Parameters to Optimize Clinical Outcomes in Trapezio-Metacarpal Prosthesis Placement Using CT Imaging, with 1-Year Follow-Up

**DOI:** 10.3390/jpm14060585

**Published:** 2024-05-29

**Authors:** Eleonora Piccirilli, Matteo Primavera, Chiara Salvati, Francesco Oliva, Umberto Tarantino

**Affiliations:** 1 Department of Orthopaedics and Traumatology Policlinico Tor Vergata, University of Rome “Tor Vergata”, 00133 Rome, Italy; matteo.primavera91@gmail.com (M.P.); chiarasalvati95@yahoo.it (C.S.); umberto.tarantino@uniroma2.it (U.T.); 2 Department of Clinical Sciences and Translational Medicine, University of Rome “Tor Vergata”, Via Mont-Pellier 1, 00133 Rome, Italy; 3 Department of Sport Traumatology, Università Telematica San Raffaele, 00166 Rome, Italy; olivafrancesco@hotmail.com

**Keywords:** TMC arthrosis, TMC joint prothesis, thumb CT

## Abstract

Purpose: Addressing trapezio-metacarpal (TMC) osteoarthritis often involves considering TMC joint replacement. Utilizing TMC prostheses offers advantages such as preserving the thumb length and more accurately replicating the thumb’s range of motion (ROM). TMC prostheses have an intrinsic risk of dislocation and aseptic loosening. Analyzing pre- and postoperative imaging can mitigate complications and improve prosthetic placement, providing insights into both successes and potential challenges, refining overall clinical outcomes. Materials and methods: We conducted a prospective analysis of 30 patients with severe TMC arthritis treated with a Touch© (Kerimedical, Geneva, Switzerland) prosthesis in 2021–2023: X-ray and CT protocols were developed to analyze A) the correct prosthesis placement and B) its correlation with clinical outcomes (VAS, Kapandji and QuickDASH scores) by performing Spearman correlation analysis. Results: The average differences in trapezium height and M1-M2 ratio pre- and post-surgery were, respectively, 1.8 mm (SD ± 1.7; *p* < 0.001) and 0.04 mm (SD ± 0.04; *p* = 0.017). Pre-to-postoperative M1 axis length increased by an average of 2.98 mm (SD ± 3.84; *p* = 0.017). Trapezial cup sinking, indicated by the trapezium index, measured 4.6 mm (SD ± 1.2). The metacarpal index averaged at 11.3 mm (SD ± 3.3). The distance between the centers of the trapezium distal surface and the prosthesis cup was 2.23 mm (SD ± 1.4). The Spearman correlation analysis gave the following results: negative correlations were highlighted between postoperative VAS scores and the M1/M2 ratio and residual trapezium height (correlation coefficient: −0.7, *p* = 0.03 and −0.064, *p* = 0.03, respectively) at 6 months; a negative correlation was found at the 3-month mark between QuickDASH and the trapezium residual height (correlation coefficient: −0.07, *p* = 0.01); and a positive correlation was found for the trapezium index at 1 month (correlation coefficient: 0.07, *p* = 0.03) and 3 months (*p* = 0.04) using the Kapandji score. Similarly, we found a positive correlation between the distance between the prosthesis and trapezium centers and QuickDASH score at 1 and 3 months (correlation coefficient: 0.066, *p* = 0.03; correlation coefficient: 0.07, *p* = 0.05, respectively) and a positive correlation between prosthesis axis and the residual first metacarpal angle with QuickDASH score at 3 months (correlation coefficient: 0.07, *p* = 0.02). Conclusions: Pre- and postoperative systematic imaging analysis should become a method for predicting complications and guiding recovery in TMC prosthesis: CT imaging could provide us with radiographical landmarks that are intrinsically linked to clinical outcomes. Further research is necessary to fuel a protocol for the correct intraoperative TMC prosthesis implantation.

## 1. Introduction

The gold standard for TMC prosthesis is tendon interposition arthroplasty; however, this surgical technique reduces the thumb length and strength [1]. Since the 70s, TMC prosthesis has been proposed as a valid alternative, especially for patients with a high functional demand related to their occupation and/or hobby. In fact, it has been documented that TMC prosthesis leads to a faster recovery and return to work and sport activities with much more tolerable painful symptoms without the need to assume painkiller therapy. In addition, postoperative immobilization of the thumb after TMC prosthesis is not mandatory, while in arthroplasty, it is maintained for 3–4 weeks [2,3]. However, TMC prosthesis has been limited by inherently linked complications: dislocation, aseptic loosening, etc. [4]. The complication rate has been reported in the literature at around 10%-20%, depending on the studies analyzed [5]. In the last two decades, a new dual-mobility prosthetic design has been introduced to specifically decrease the risk of dislocation [6,7]. For more well-studied joint prostheses, preoperative and postoperative imaging analyses are fundamental for (A) selecting patients, (B) preoperative panning, (C) postoperative assessment, and (D) correcting pitfalls to improve the clinical outcomes and the future evolution of TMC prostheses. Patient selection has been well examined in the literature; the presence of STT arthritis and/or a trapezium height of <8 mm preclude prosthetic placement [8]. Similarly, authors have proposed anatomical parameters to be used in preoperative planning: the M1-M2 arch and M1/M2 ratio are two additional resources for preserving the thumb’s height and its ROM [9]. The literature is still lacking in studies comparing preoperative and postoperative imaging analyses to clinical outcomes. The aim of our paper is to analyze the preoperative and postoperative RXs and CTs of 30 patients treated with TMC prosthesis and analyze their influence on the planning of treatment, surgical technique, and clinical outcomes.

## 2. Materials and Methods

### 2.1. Study Design

Our prospective study includes 30 patients with Eaton–Litter stage II and III TMC osteoarthritis and treated with a Touch© prosthesis at our institution in the period 2021–2023. Demographic data are summarized in Table 1 Preoperative analysis included radiographs (in the antero-posterior position and lateral projection) and CT scans using a standardized protocol. Criteria of inclusion were failed conservative treatment, no signs of STT arthritis, a trapezium height of ≥8 mm, and patients’ high functioning. VAS scales and QuickDASH questionnaires were submitted to all patients preoperatively and postoperatively at scheduled follow-ups: 1, 3, and 6 months and 1 year. The QuickDASH test is composed of 11 items used to measure physical function and symptoms in people with any or multiple musculoskeletal disorders of the upper limb. The Kapandji score was also measured at the same time intervals. The Kapandji score is a tool that is useful for assessing the opposition of the thumb based on where on their hand the patient is able to touch with the tip of their thumb. Patient satisfaction was collected at the 6-month mark and classified as “very satisfied”, “satisfied”, or “disappointed”. We acquired the HIPAA and informed consent for all the included patients; the present study was approved by our institution’s ethical board.

### 2.2. Prosthesis Design

All 30 Touch© prostheses were implanted by the same senior surgeon; the Touch© is dual-mobility prothesis with a steel stem, neck, and metallic head all in different sizes. The trapezium cup may be in a spherical or conical shape. The modularity of the prosthesis is key in producing 144 possible configurations to fit the patients’ anatomy.

### 2.3. Surgical Technique

Following a brachial plexus block, the patient was placed in a supine position, and a high-arm tourniquet was inflated to 250 mmHg. A dorsal longitudinal incision was made over the TMC joint, followed by careful superficial and deep dissection of the TMC joint. With an oscillating saw, around 5 mm of the base of the first metacarpal was cut off first, followed by the distal surface of the trapezium. Additionally, any osteophyte was removed. Then, the prosthetic components were implanted in the following order: the metacarpal stem, the cup on the trapezium, the neck, and the head. Size selection was based on passive mobility tests and according to the surgeon’s preference. Correct thumb length was assessed intraoperatively. The capsule was sutured back; skin suturing was carried out with absorbable stiches, and the hand was treated with dressings.

### 2.4. Postoperative Management and Follow-Up

All patients underwent a standardized postoperative management protocol: the thumb was immobilized with appropriate dressings for 2 weeks; then, all 30 patients started gradual ROM exercises guided by a hand therapist. All patients received postoperative pain management and were advised to avoid heavy lifting, sport, or strenuous activities during the initial recovery phase. Follow-up evaluations were scheduled at regular intervals at 1, 3, and 6 months and at 1 year postoperatively and included VAS, Kapandji, and QuickDASH scores assessments and X-rays.

### 2.5. Imaging Analysis

**Figure 1:**
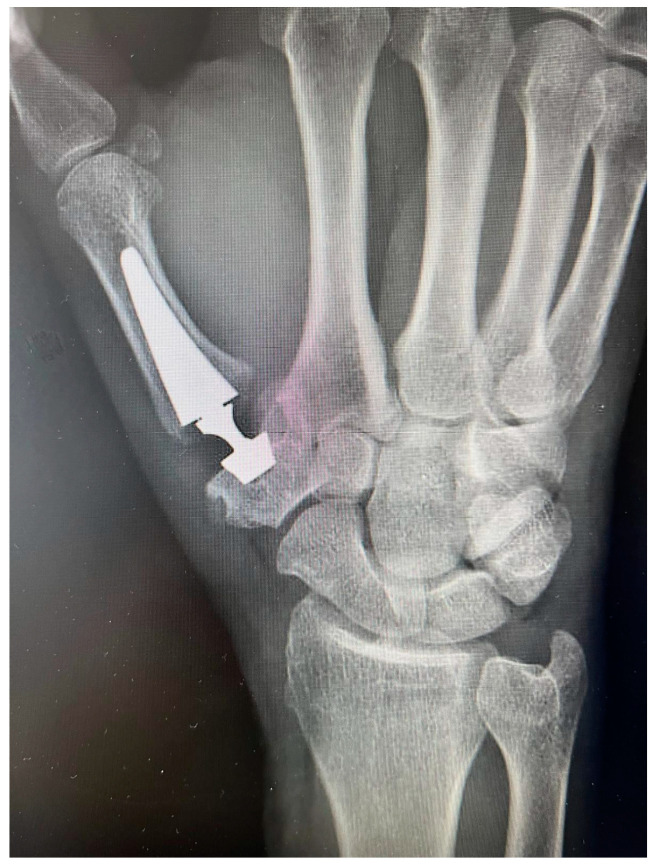
Trapeziometacarpal prosthesis Touch© (Kerimedical).

**Figure 2:**
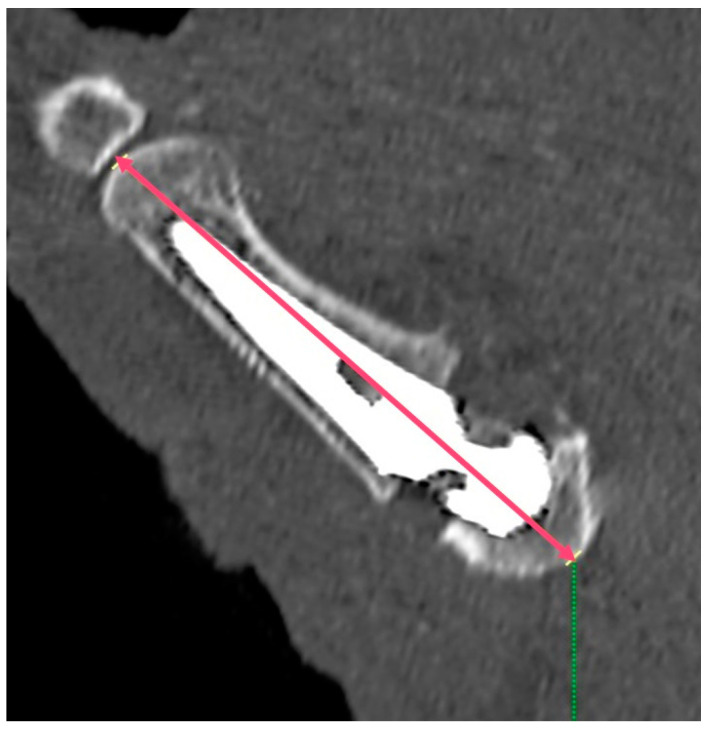
The postoperative M1 axis, defined as the length of the first metacarpal and the trapezium combined.

**Figure 3:**
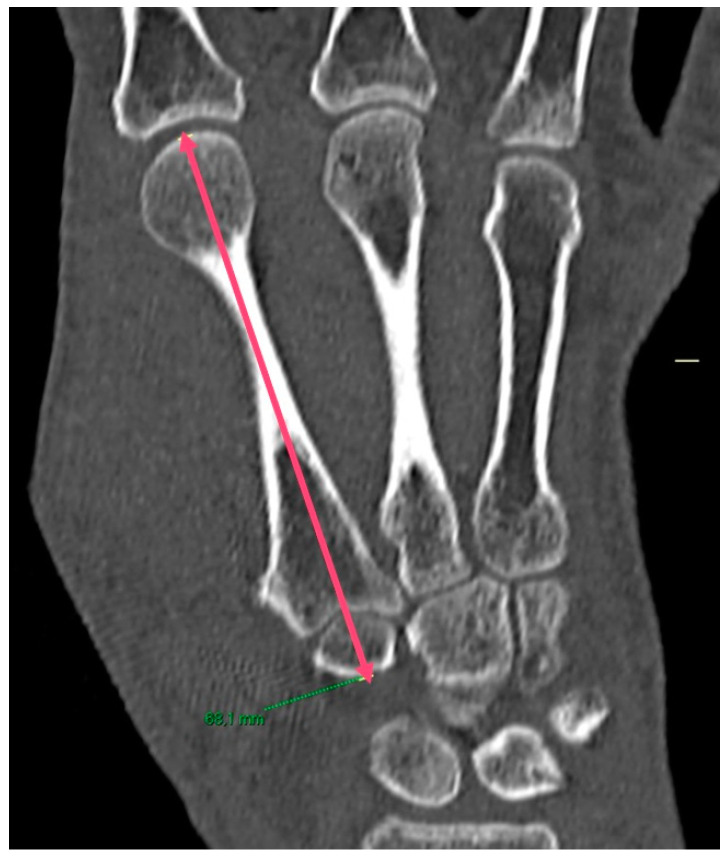
The M2 axis, defined as the length of the second metacarpal and the trapezoid bone (red line).

**Figure 4:**
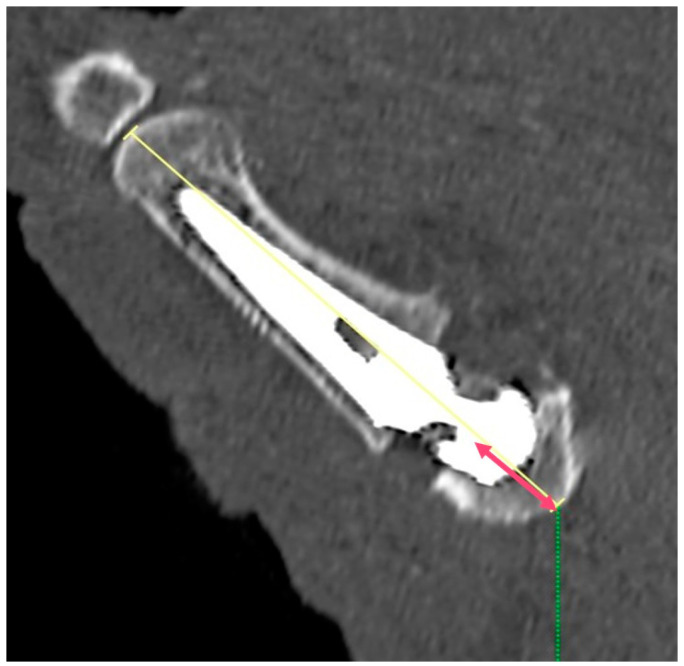
Trapezium height, calculated pre- and postoperatively in the coronal axis from the proximal to the distal surface of the trapezium bone.

**Figure 5:**
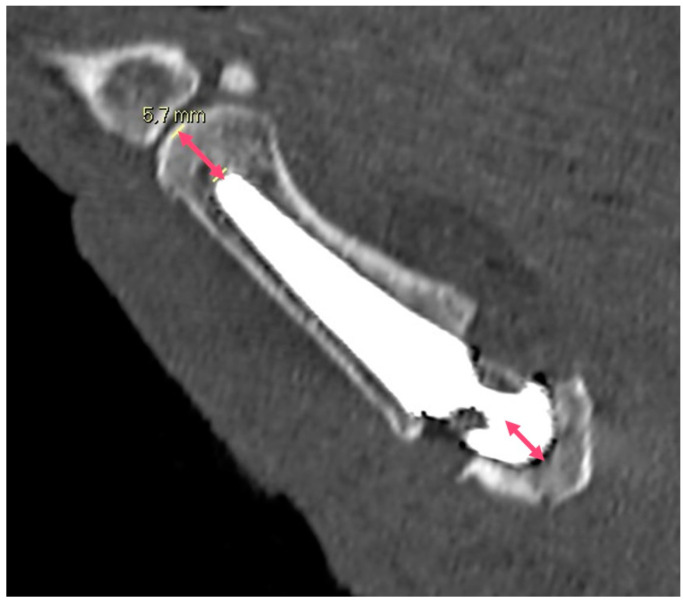
Metacarpal index (defined as the space of the first metacarpal not occupied by the prosthesis stem) and the trapezium index (defined as the space of the trapezium bone occupied by the cup); both are calculated in the coronal plane.

**Figure 6:**
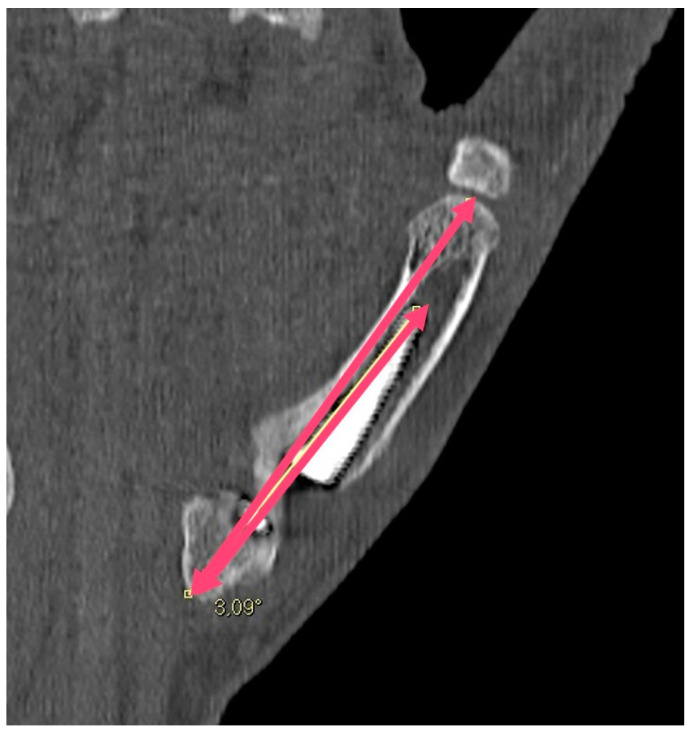
The angle between the M1 axis (as defined above) and the prosthesis axis (shorter red line), measured in order to study the proper alignment of the two structures.

**Figure 7:**
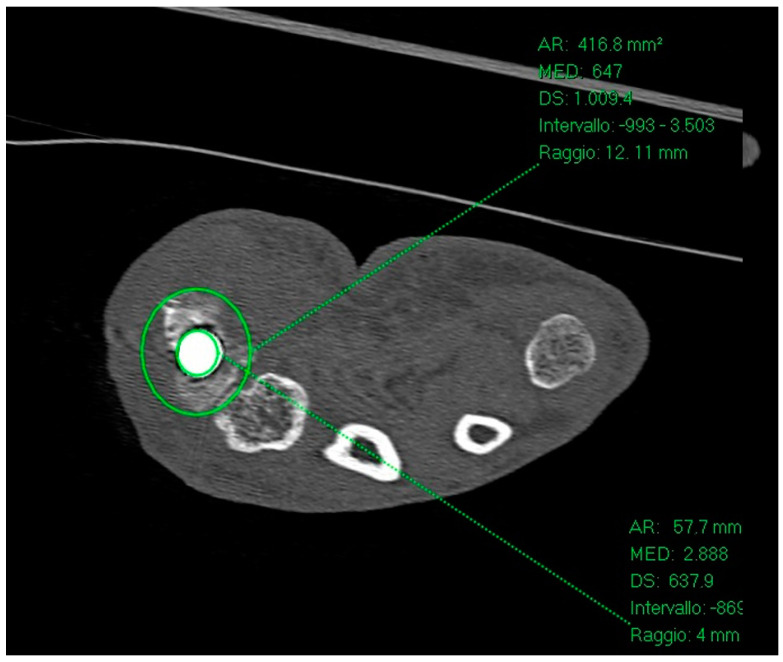
The distance between the center of the trapezium bone and the cup prosthesis can be studied in the sagittal view by drawing two concentric circles (one based on the trapezium and the other on the prosthesis cup) and then measuring the distance between their respective centers.

Preoperative X-rays were used to identify deformities and for TMC arthritis staging. CT imaging was carried out preoperatively according to our internal protocol, which was developed with the aid of the Radiology Department of our center and had the following schedule: 1 week preoperatively and within 24 h from the procedure. Control X-rays (Figure 1) were also carried out 1, 3, and 6 months and 1 year following prosthesis implantation. CTs were collected and analyzed with a 256-layered GE Revolution CT scanner (Figure 2, Figure 3, Figure 4, Figure 5, Figure 6 and Figure 7). All parameters were independently assessed by two different radiologists using only the CT imaging and are reported in Table 2 The parameters were defined as follows: (1) preoperative and postoperative trapezium height; (2) M1 length measured as a central axis starting from the distal surface of the first metacarpal and extending to the proximal articular surface of the trapezium; (3) M2 length recorded a central axis starting from the distal articular surface of the second metacarpal to the proximal articular surface of the trapezium; (4) M1/M2 ratio as theorized by Ledoux et al. [9]; (5) the difference between the pre- and postoperative M1/M2 ratio; and (6) the M1 axis; (7) the angle between the axis of the prosthesis (a line passing from the tip of the stem to the center of the trapezial cup) and the central axis of the first metacarpal; (8) the angle between the trapezial cup distal surface and the central axis of the trapezium to calculate the right cup placement; and (9) the distance between the center of the cup and center of the trapezium calculated at the transverse section.

### 2.6. Statistical Analysis

The analysis was performed using Excel™ (Microsoft, Redmond, WA, USA): descriptive statistics were used to summarize the data, including means, standard deviations, and percentages. The Shapiro–Wilk test was used to assess the normal distribution of data. Spearman correlation analysis was employed, as the data were not normally distributed, to investigate potential associations between various radiological parameters and the clinical outcomes. Significance was set at a *p*-value < 0.05. This study was conducted in compliance with the principles outlined in the Declaration of Helsinki.

## 3. Results

### 3.1. CT Results

The average difference between the pre- and postoperative trapezial height was 0.8 mm (SD ± 1,2; *p* value < 0.001). The difference between pre- and postoperative M1/M2 values was 0.04 mm (SD ± 0.04; *p*-value = 0.017); the difference in length between the pre- and postoperative M1 axis alone showed an average increase of 2.61 mm (SD ± 3.12; *p*-value = 0.017). The sinking of the trapezial cup was measured with the trapezium index (4.5 mm; SD ± 0,1) while the average metacarpal index was of 11.3 mm (SD ± 3.3). The distance between the center of the trapezium and the center of the prosthesis cup was 2.2 mm (SD ± 1.3). All measurements are summarized in Table 2.

### 3.2. Correlation with Clinical Results

Clinical outcomes are summarized in Table 3 and analyzed with Spearman correlation analysis: specifically, a negative correlation emerged between postoperative VAS scores and the difference between pre- and postoperative M1/M2 ratios (correlation coefficient: −0.7, *p*-value: 0.03). Additionally, a significant negative correlation was observed between VAS scores and residual trapezium height (correlation coefficient: −0.064, *p*-value: 0.03); residual trapezium height also had a significant negative correlation at the 3- month mark according to QuickDASH score (correlation coefficient = −0.07, *p*-value: 0.01). Regarding the Kapandji score, a positive correlation of 0.07 with the trapezium index was recorded at 1 month (*p*-value = 0.03) and 3 months (*p*-value = 0.04). The distance between the centers of the prosthesis and the trapezium showed a positive correlation with QuickDASH score at 1 and 3 months (correlation coefficient = 0.066, *p*-value = 0.03 and correlation coefficient = 0.07, *p*-value = 0.05, respectively). The angle between the axis of the prosthesis and the residual first metacarpal had a positive correlation with QuickDASH score at 3 months (correlation coefficient: 0.07, *p*-value: 0.02). No significant correlation could be observed between the radiographical measurements alone. No significant correlation was found at 1-year follow-up. All Spearman correlation analysis results are summarized in Table 3.

## 4. Discussion

Pre- and postoperative radiographic analysis is essential for improving knowledge of prosthetic placements that are commonly carried out during knee and hip replacement surgery; our group found no studies that directly correlate radiological findings with clinical outcome for TMC prosthesis. Additionally, when describing TMC prostheses’ radiological parameters, papers are not homogenous in their definitions, nor are they consistent on whether they are useful in clinical practice. For preoperative planning, the consensus is to assess trapezium height (which should be of at least 8 mm [8]) and identify osteophytes and deformities of the first ray. Postoperative analysis is mainly focused on the prosthesis loosening, migration, and eventual dislocation. Exploring the correlation between radiographic landmarks and the clinical outcome for TMC prosthesis has various advantages: (1) it can potentially reduce the risk of mispositioning or misalignment by making intraoperative corrections; (2) it may enable customization of prosthesis placement based on individual patient anatomy and needs; and (3) it is a learning tool for optimizing prosthesis implantation. When compared to X-rays, CT imaging, while not mandatory for every TMC arthritic patient, is important in the context of research for achieving more precise measurements. CT scans were employed to measure parameters already know in the literature, such as the M1/M2 ratio and the metacarpal and trapezium indexes [10], as well as newly introduced ones: (1) pre- and postoperative differences in the length of M1; (2) the M1/M2 ratio; (3) the angle formed by the axis of M1 and the prosthesis; (4) the angle formed by the cup and a line perpendicular to the trapezium proximal surface; and (5) the distance between the center of the trapezium and the center of the cup, taken with a CT transverse section. In particular, we excluded the M1-M2 arch (as theorized by Ledoux [9]) as more difficult to quantify for the purpose statistical correlation analysis, even if it is a useful intraoperative parameter for confirming the definitive restored thumb length. Our data regarding the difference in first metacarpal length (pre- and postoperatively) match the ones present in the literature as the M1/M2 ratio [6,9,11,12,13]. In our study the M1/M2 postoperative ratio was negatively correlated with the VAS score at 6 months (*p* = 0.03); this could demonstrate that by increasing the length of the M1, we may cause a decrease in perceived pain. However, the beneficial range of M1 axis increase is difficult to quantify, and we did not find a direct significant correlation with postoperative M1 length. Regarding the trapezium, its preserved postoperative height correlates with a lower VAS score at 6 months and QuickDASH score at 3 months: both factors likely protect the biomechanics of the thumb tendons (which also aligns with our observed lack of postoperative DeQuervain’s tenosynovitis, in contrast to other studies [14]). The metacarpal and the trapezium indexes have been studied to monitor the possible dislocation of prostheses; our recorded values are in line with those in the literature, while the trapezial index has been found to positively correlate with the Kapandji score at 3 months. This could be attributed to the trapezial index quantifying cup insertion and osteointegration, both of which are crucial for prosthesis stability during the rehabilitation phase; both the trapezium and metacarpal indexes should be analyzed for longer follow-up [6]. Our group showed correlations in two new measurements: (1) the angle between the central axis of the Touch© prosthesis and the axis of the first metacarpal and (2) the distance between the center of the prosthesis and the center of the trapezium. Both correlated positively with QuickDASH score at 1 month, while the distance between the center also correlated at 3 months. A reduction in this angle could reflect a more precise placement of the stem, paralleling the natural first metacarpal axis. Finally, our results stress the importance of centering the cup on the trapezium as this step can be tricky to optimize intraoperatively but is essential to limit cup migration risk [15,16]; reducing the distance between the center of the cup and the trapezium translates in a more stable cup and a decrease in QuickDASH score. In the postoperative phase, any cup migration and/or loosening has to be to analyzed for any defective functioning of the thumb, as already pointed out by previous studies [17,18,19]. The limits of our study include the small number of patients included and the short follow-ups.

Systematic pre- and postoperative imaging analysis could help in predicting complications and guiding patients’ recovery. The VAS score is an important tool for modulating painkilling medications for successful rehabilitation; both the QuickDASH and the Kapandji scores are linked with proper prosthesis placement and first ray length restoration, which in turn influence patients’ daily activities. Future studies could focus on comparing some of the above proposed measurements with TMC prostheses and arthroplasties, featuring longer follow-ups. Our paper encourages the adoption of internal protocols to check the proposed radiological landmarks, with emphasis on the ones that showed a statistically significant correlation: the distance between the centers the trapezium and the cup (better if analyzed using CT), the angle between the axis of the prosthesis and the first ray, and the already proposed trapezium index and the postoperative M1/M2 ratio (always to be compared with preoperative measurements). We can conclude that a correct preoperative radiographic evaluation is crucial for correct planning to optimize the prosthetic implant technique. The objective is to standardize implants with future dedicated protocols to minimize the risks of loosening and dislocation of prostheses.

## Figures and Tables

**Table 1:** Patients’ characteristics.

	N	Average	Standard Deviation
W	17		
M	13		
AGE	-	59.2	10.8
Right side affected	18		
Left side affected	12		

**Table 2:** CT-derived parameter measurements.

Parameter	Average	Standard Deviation
PreOP trapezium height	11.0 mm	±2.8 mm
PostOP trapezium height	10.1 mm	±1.7 mm
PreOP M1 height	54.9 mm	±4.5 mm
PostOP M1 height	57.9 mm	±3.9 mm
PostOP M2 height	74.0 mm	±5.2 mm
PreOP M1-M2 ratio	0.74	0.06
PostOP M1-M2 ratio	0.78	0.02
Difference between Pre and PostOP M1-M2 ratio	0.04	0.04
M1 height difference PreOP and PostOP	2.9 mm	±3.2 mm
Angle between the prosthesis axis and the M1 axis	2.8°	±1.3°
Metacarpal index	11.3	±3.3
Trapezium index	4.5	±0.1
Angle between the cup distal surface and the trapezium	2.0°	±3.7°
Distance between the center of the trapezium and the center of the cup	2.2 mm	±1.3 mm

**Table 3:** Clinical outcomes.

Parameter	Average	Standard Deviation
PreOP VAS	7.4	±1.3
PostOP VAS at 1 month	5.2	±0.8
PostOP VAS at 2 months	2.1	±0.5
PostOP VAS at 6 months	0.5	±1.0
PostOP VASat 1 year	0.5	±0.3
PreOP QuickDASH	72.3	±15.1
QuickDASH at 1 month	45.8	±5.5
QuickDASH at 3 months	10.5	±14.8
QuickDASH at 6 months	1.8	±4.3
QuickDASH at 1 year	1.9	±1.8
PreOP Kapandji score	6	±0.8
Kapandji score at 1 month	7.7	±1.1
Kapandji score at 3 months	8.1	±0.3
Kapandji score at 6 months	9.8	±0.4
Kapandji score at 1 year	9.8	±0.3

## Data Availability

Available data are contained in tables in in the manuscript.
